# Epistemological Foundation for the Use of the Linguistic Measures of the Referential Process

**DOI:** 10.1007/s10936-025-10149-1

**Published:** 2025-05-08

**Authors:** Attà Negri, Arianna Barazzetti

**Affiliations:** Department of Human and Social Sciences, University of Bergamo, Piazzale S. Agostino 2, Bergamo, 24129 Italy

**Keywords:** Multiple code theory, Referential process, Referential activity, Reflection and reorganization function, Complexity epistemology

## Abstract

The analysis of epistemological beliefs underlying psychotherapeutic interventions has been largely neglected by research in psychotherapy, training in psychotherapy and psychology, and often by theorists of different clinical orientations. The main risks of this neglect are the unexamined adoption of the epistemology that is taken for granted by the culture and is largely inconsistent with the specifics of the object of study of psychological science; the reduction of intervention effectiveness due to the inconsistency between epistemology, theory, and practice; and the maintenance of the gap between research and practice due to the different epistemologies used by clinicians and researchers. This article discusses the scientific status of the computerized linguistic measures of the Referential Process when used for clinical and research purposes. Our claim is that these measures developed to test Wilma Bucci’s multiple code theory don’t represent an objective examination of the psychotherapeutic process, but rather a methodological option to guide the researcher and clinician in identifying the most plausible scientific hypotheses about the complex phenomenon of emotional communication between speakers. Comparison of data from different points of view (therapist, patient, external observer, computerized linguistic analysis, etc.) and in different contexts (therapies, psychological tests, everyday conversations, experimental situations, etc.) will be presented as promising and viable ways to examine the validity of the hypotheses based on the Referential Process theory.

## The Need for an Appropriate Epistemological Foundation in Psychology

Before any scientific knowledge endeavor, it seems necessary to define and agree on what are the necessary and sufficient conditions for the research process to produce reliable, that is scientific, knowledge. In other words, it appears necessary to outline an epistemological theory, a theory that delimits the specific object of scientific investigation, defines how it is possible to know it reliably, and indicates how to act on it effectively.

If this is true for any scientific domain, it is even more true for those scientific domains where the objects of investigation are immaterial such as, for example, those of psychology, sociology, and philosophy, where the objects of investigation exist only as mental and cultural artifacts, thus, they exist insofar as people think they exist. Even scientific domains whose objects are material but intangible, i.e., only indirectly investigable such as atomic and subatomic physics for example, need an a priori epistemological theory that is foundational to the knowledge process itself. However, the scientific field that requires an epistemological foundation more than others is psychology, especially when it is understood as the study of the human mind and subjectivity, rather than merely the study of observable behaviors. The human mental processes of subjective experience are not only immaterial, intangible, and particularly complex, but they are knowable only through a process of encountering the subjectivity of the investigator with the subjectivity of the one being investigated. In other words, in psychology human subjectivity is both the object of study and the instrument of knowledge; in this scientific realm, therefore, it is crucial to rigorously and explicitly define the epistemological coordinates that guide the scientific knowledge and professional intervention (Blasi, 2018; Salvatore & Ruggeri, 2018).

The epistemology not only defines the reliability of the knowledge produced by the scientific efforts but also ensures the effectiveness and appropriateness of the interventions that derive their justification from it. In fact, the epistemological theory underlying a scientific process defines the constraints and possibilities within which to choose the most appropriate reference theories about the object of investigation. In turn, these theories determine the relevant and justified objectives to be set in studying and intervene on that object, as well as the technical means that are relevant and justified to achieve those objectives. In other words, theoretical, methodological, and technical decisions stem directly from the epistemological level. If for example we remain in the field of psychology, adopting a positivist epistemology will lead us to choose theories that conceive of the mind as a set of elements that belong to the individual, are externally knowable, and tend to be objectifiable. In this context, we will also be inclined to think of psychological interventions as actions that externally and unilaterally act on the causes of certain effects, adopting intervention protocols that tend to be standardized and technical to achieve the desired and a priori expected effects. In sum, we will treat the object of study and intervention similarly to material objects that are directly knowable and modifiable from the outside (Negri et al., 2019a, b).

The scientific revolution that occurred in the 16th and 17th centuries, the subsequent Enlightenment^1^ movement, and the industrial revolution of the 19th century laid the foundation for the development in the cultures of the Western world of a conception of science based on a positivist epistemology. This perspective is based on a mechanistic conception of nature, on the reduction of phenomena to their constituent elements and causes, on predictions based on fixed relationships between causes and effects, on the discovery of the working laws of phenomena, and, above all, on the limitation of subjective influence in the process of knowledge of which the laboratory experiment is the emblematic example (Kramer et al., 1992). This epistemology developed and was assumed in an automatic and unaware way so that for a long time there was never any talk of the epistemology of science; even today, the prevailing belief is that the scientific validity of knowledge is guaranteed by the experimental method, rather than by defining the epistemological framework that underpins knowledge, including the experimental method itself. This positivist epistemological conception led to an enormous advancement of knowledge in the so-called “nature sciences” or “hard sciences” that gradually opposed the “spirit sciences” or “soft sciences.” In fact, in the latter, such a conception of science was not applicable and thus they accrued an inferior or rather delegitimized status in the dominant scientific culture (Ceruti, 2015).

Psychology, which among the sciences is one of the youngest, has not been unscathed by the allure of automatically assuming, from the very beginning, the positivist epistemology as the basis for its recognition as a science in the Western cultures. This has led to theories that equate the mind with tangible, material objects and assume the experimental method as the paradigm of “scientificity,” even though this leads to a considerable and critical reduction – Hoffman (2009) would speak of desiccation – of the characteristic and distinctive properties of its object, namely, human subjectivity. Failing to adapt the epistemological conception to the new object of inquiry also led Freud to brilliant but partial and inconsistent discoveries. Indeed, it is now widely pointed out that explaining the mind in terms of somatopsychic energies and drives greatly reduces the heuristic scope of Freud’s brilliant clinical hypotheses (Assoun, 1981; Bucci, 1989). But even currently, psychological science remains predominantly rooted in a positivist view of science, with the epistemological assumptions underlying its knowledge and practices largely remaining unexamined. The main theoretical and research currents offer explanations of mental functioning in terms of visible behaviors or neurobiological circuits, descriptions of phenomena rather than theories, standardized intervention protocols, and evidence-based procedures where “evidence” is what reduces and bypasses the complexity of subjective and intersubjective phenomena.

The lack of attention in psychology to epistemological beliefs underlying knowledge and interventions carries several significant risks, including the unexamined adoption of the epistemology taken for granted by our culture; the adoption of epistemologies that may not align with the specific nature of the object under study or intervention; a potential decrease in intervention effectiveness due to inconsistencies between epistemology, theory, and practice; and the perpetuation of the gap between research and practice resulting from clinicians and researchers operating under different epistemological frameworks (Fourie, 1996; Negri et al., 2019a ).

In the field of clinical psychology and psychotherapy, a paradigm shift toward what is termed a complexity-oriented epistemology has only recently and partially taken hold among practitioners and researchers. It refers to an approach that embraces the inherent complexity of human subjectivity and interactions, recognizing that human experiences cannot be fully understood through reductionist methods alone. It advocates for a more integrative perspective, acknowledging the dynamic, interconnected, and often non-linear nature of psychological phenomena (Ryan & Deci, 2006). Notable examples of this paradigm shift include the emergence of intersubjective and relational theories in psychoanalysis (Aron, 1996; Mitchell, 1988; Stolorow & Atwood, 1992), the post rationalist turn in cognitivism (Guidano, 1987, 1991; Guidano & Liotti, 1985), 10.1007/978-1-4684-7562-3_4 and the transition to second-order cybernetic theories in systemic therapy (Bateson, 1972; Maturana & Varela, 1980; Ugazio, 2013; Von Foerster, 1987). While the development of new research methods aligned with complexity epistemology is ongoing, approaches like single case studies and systematic analyses of contextualized situations show promise for capturing human complexity (Hayes, 1981; Hoffman, 2009; Kazdin, 1981; Wall et al., 2017).

The aim of the present work is to propose and discuss the epistemological foundation of the Multiple Code Theory (MCT; Bucci, 1997a, 2021) as a general theory of mind based on a complexity-oriented epistemology and consistent with the properties of the object of investigation: human subjective experience and emotional communication. Furthermore, the hypothesis is that the computerized linguistic measures developed to apply Wilma Bucci’s multiple code theory represent not an objective examination of the psychotherapeutic process, but a methodological option to guide the researcher and clinician in identifying the most plausible scientific hypotheses about the complex phenomenon of emotional communication between speakers.

## Epistemologies in Comparison

Drawing upon Schwandt’s (2001) conceptualization of epistemology as the “study of the nature of knowledge and justification” (p. 71), we define epistemology as a set of assumptions about the nature of scientific knowledge, the conditions that underly its acquisition and development, the models of explication adopted, the theories of influence between the subject and object of the knowledge process, and the criteria for choosing the more plausible hypotheses about the reality under investigation (Carson et al., 2001).

In the literature, there are several descriptions and classifications of the epistemological positions that can be detected underlying scientific research and professional intervention (for example, Berzonsky, 2016; Felton & Kuhn, 2007; Hofer, 2000; Kramer et al., 1992). Here, we want to highlight two opposite extremes of a continuum of various possible epistemological positions; in the field of psychology, these extremes can have radically different effects on the choice of the theories of mind, methods of intervention, and techniques adopted. On one extreme, we have placed the “positivist epistemology,” and on the other, the “complexity epistemology” (see Table 1). We have organized the comparison along three dimensions: the objectivity-subjectivity dichotomy, focusing on the influence of the observer; the certainty-uncertainty dichotomy, concerning the nature of knowledge; and the explanatory models used in terms of causality and modes of argumentation. These themes were selected because they represent the fundamental differences between the two approaches.

**Table 1:** Principal differences between the positivist and complexity epistemologies

Positivist Epistemology	Complexity Epistemology
*Dichotomy Objectivity-Subjectivity*	
Reality is explainable by general rules that can be objectively discovered	Reality is explainable by multiple tentative assumptions that may or not may be intersubjectively validated
Reality is knowable directly, objectively, and independently of the observer’s subjectivity	Reality is knowable indirectly, subjectively, and through observers’ interactions and contextual influence
The subjectivity of the observer limits the knowledge	The subjectivity of the observer is an essential component of the knowledge
The laboratory experiment is a model that ensures objective knowledge by controlling subjectivity and manipulating variables	Multiple intersubjective validation by a plurality of viewpoints and multiple levels of analysis allows for more valid knowledge
*Dichotomy Certainty-Uncertainty*	
Reality is characterized by simplicity, rationality, and causality	Reality is characterized by complexity, non-linearity, and emergence
Only clear, certain, and empirically verified statements are accepted	Multiple hypotheses can coexist, and uncertainty is inherent in knowledge
Scientific knowledge proceeds by accumulation of scientific truths	Scientific knowledge involves paradigm shifts
*Explicative models*	
The external, unidirectional, and linear influence on reality is possible	The influence on reality is bidirectional, circular, and always from within
Linear causal explanation models are sought to make certain predictions that link causes and effects	Circular causal explanation models are sought to make probabilistic inferences about multiple possible trajectories
Causes are explanatory of phenomena	Reasons are explanatory of the phenomena
The approach follows the principle that the simpler explains the more complex	The approach follows the principle that the more complex explains the simpler

The positivist epistemology posits that reality exists independently of the observer and advocates for the direct and objective discovery of truth through the application of scientific methods, as expounded by Casti (1989). Under the auspices of positivist epistemology, scientific inquiry is directed towards the accumulation of new discoveries built upon prior ones, with the ideal trajectory towards omniscience, as expounded by Ceruti (2015). On the other hand, in the complexity epistemology, reality is not viewed as static or pre-determined, but rather dynamically constructed through interactions within systems and influenced by environmental factors (Fuchs, 2010). This suggests that reality is not uniform but rather contingent upon context and perspective.

The complexity approach acknowledges the subjectivity of the observer’s perspective and beliefs, recognizing that interpretations of reality may vary based on individual experiences and viewpoints; it embraces uncertainty and multiple viewpoints, suggesting that knowledge is constructed through the integration of diverse perspectives. Conversely, the positivist approach emphasizes objectivity and empirical verification, disregarding the observer’s subjective views in favor of uncovering universal and certain truths through systematic observation and experimentation.

Regarding the explicative models adopted, the complexity approach researches the intricacies and interconnectedness of phenomena, recognizing that reality is characterized by complexity, non-linearity, and emergence. In contrast, the positivist approach seeks to uncover universal laws and principles explaining the reality viewed as predictable and explainable by logical relationships and cause-effect mechanisms.

Psychology, psychotherapy, and the social sciences have often unknowingly assumed the dominant positivist epistemology (Assoun, 1981; Robertis, 1997; Watson, 1913). For instance, behaviorism defined psychology as an objective experimental science aimed at predicting and controlling behavior, while even Freudian metapsychology, despite contradictions, adopted a positivist view, treating psychoanalysis as a natural science (Assoun, 1981; Robertis, 1997). Similarly, early systemic orientations leaned towards a positivist phase, seeking objective views of system functioning (Watzlawick et al., 1967).

These epistemological positions have implications on the grounds of the theory of clinical intervention, the theory of mind adopted, and the techniques employed in both clinical practice and research as summarized in Tables 2 and 3.

In comparing positivist and complexity epistemologies within the context of psychotherapy, we summarized in Table 2 the main significant implications they have in many aspects such as the positions of the patient and therapist (external vs. internal; expert vs. receiver), the main object of intervention (behaviors vs. meaning-making process), theory of mind (recipients of entities vs. connecting system), explanatory models (cause-and-effect mechanisms vs. complex interactions), diagnostic process (descriptive vs. explanatory), and goals of therapy (transforming patients vs. creating a working relationship).

**Table 2:** Implications of assuming a positivist or complexity epistemology for the theory and practice of psychotherapy

Positivist Epistemology	Complexity Epistemology
*Therapists’ role*	
Experts of the individuals and their symptoms and functioning; external observers, focusing on prediction and control of behavior and mental processes	Experts in reading and activating the resources of the patient relationship; co-creators of the therapeutic exchange, engaging patients in a process of meaning-making and change
*Patients’ role*	
Recipients of diagnoses and treatments of which they are not the experts	Potential experts and agents of their change within the relationship with the clinician
*Theory of mind and focus of the interventions*	
Mind as a set of internal and individual states, with a focus on stable traits and observable behaviors	Mind as a set of connecting ways with the context, an open and dynamic system consisting of processes of signification influenced by multiple factors and contexts
*Causal relationships*	
Typically viewed as linear and unidirectional, emphasizing cause-effect mechanisms	Recognized as multiple, circular, and context-dependent, acknowledging complex interactions between elements and events
*Diagnosis*	
Nomothetic and descriptive diagnosis of symptoms and personality, treating mental health issues as stable entities	Diagnosis of relational patterns and processes (including those emerging in the therapeutic relationship), focusing on contextual, idiosyncratic, and consensual understanding of patients’ experiences
*Psychotherapist’s professional aims*	
Transforming patients from a current state to a priori predefined desirable states using standardized and transformative techniques	Creating a relationship with patients that activates in them their resources and processes of change and development that cannot be predicted in advance
*Professional status*	
Clinicians’ competences are those assigned to them by culture, and they are similar to those assigned to the medical professionals who are Experts in disease and treatment	Clinicians’ competencies are different from those ascribed to them by patients and culture and they must be renegotiated in accordance with the chosen theory and epistemology
*Aims of techniques and tests*	
Techniques and tests are tools for an objective investigation of personality and changes; they investigate the individuals from an external point of observation; the standardization limits the subjectivity	Techniques and tests are tools for creating the relationship with patients and for eliciting different aspects of the subjectivity investigated from an internal point of view; standardization assures the comparison between different individuals’ experiences

In Table 3, we summarized the implications for research in psychology and psychotherapy as in the other applied fields that the two epistemological extremes have. The main differences include the role attributed to the subjectivity (limit vs. means of knowledge), the assurance of the achievement of scientific knowledge (experimental method vs. intersubjective tally), the privileged strategy of analysis (molecular vs. molar), and the nature of scientific findings (absolute and permanent vs. contextual and temporary).

It is important to acknowledge that while here we offer a clear distinction between positivist and complexity epistemologies in psychotherapy and research, these classifications are ideal types. Practices often fall along a continuum between these two approaches, with varying degrees of adherence to each paradigm or to both in a contradictory way.

**Table 3:** Implications of assuming a positivist or complexity epistemology for the research process

Positivist Epistemology	Complexity Epistemology
*Procedures*	
Uses standardized artificial settings such as laboratory experiments and randomized clinical trials to get a reliable and objective knowledge	Uses flexible, reflexive, and intersubjective practices within the ecological contexts to grasp a plausible and situated knowledge
*Strategies*	
Aimed at controlling variables, limiting subjective influence and bias, ensuring replicability, and breaking down phenomena into their basic and simple elements that explain them	Aimed at investigating the interaction of the multiple variables including the researchers influences, valuing the uniqueness and subjective point of views, broadening the observation field to systemic explanations
*Hypotheses testing*	
Assured by methods and procedures, it consists of checking the correspondence between hypotheses and reality by adding elements to previously verified knowledge	Assured by intersubjective tallying on the same reality, it consists of establishing criteria for choosing the more plausible hypotheses, which always remain temporary and tentative

## Multiple Code Theory and Complexity Epistemology

The Multiple Code Theory (MCT), developed by Bucci (1997a, 2021a), presents an encompassing framework elucidating the processing of emotional information and the functions implicated in psychotherapeutic change. Grounded in advancements in cognitive psychology, psychoanalysis, and affective neuroscience, MCT hypothesizes the presence within individuals of at least three different coding systems and formats: (a) the subsymbolic system that processes the continuous flow of emotional and sensory stimuli in global and analogical modes, operating along continuous dimensions and without discrete elements or units, managing multiple pieces of data concurrently; (b) the nonverbal symbolic system that creates images and representations from the ongoing flow of subsymbolic experience; and (c) the verbal symbolic system that connects and translates these images and representations into words.

Hypothesizing these three processing systems, MCT considers the mind not as a set of internally fixed states, but as an ongoing relational process between the subject and the world. Moreover, it recognizes that the mind is intrinsically embodied. This perspective challenges the traditional notion of a separate and distinct mind from the body, instead emphasizing the integral role of bodily experiences in cognition. By acknowledging the body as a fundamental component of mental processes, MCT embraces a holistic understanding of the mind that transcends the confines of the brain and extends into the realm of embodied experience. Supporting the idea that the mind is both enactive and embodied offers us a richer comprehension of how knowledge is formed, perceived, and lived—an outlook that resonates with the fundamental tenets of complexity epistemology. In this context, the notion of the mind takes on a new perspective, characterized as a system for organizing and assigning meaning that draws from both subtle, non-verbal experiences and the ability to communicate through symbols (Bucci, 1984, 1997a, 2021a). The mind extends beyond symbolic representations to encompass subtler, non-verbal aspects of experience. It is the ongoing interplay between these two layers of functioning that enables individuals to comprehend their reality and consequently, to engage with it.

A second central point of Bucci’s theory is the concept of Referential Process (RP) (2021b; Bucci et al., 2016), defined as the function of connecting the continuous and complex flow of emotional and sensory experience to mental images and linguistic expressions, thus making it communicable to others. This process remains partial, as not all emotional and sensory experience can be fully connected to images and words. As a result, a certain degree of disconnection persists between individuals’ feelings and their ability to symbolize and convey them to others. Furthermore, when individuals undergo distressing events, emotions or relationships, certain mechanisms geared towards alleviating and suppressing painful sensations may be triggered automatically. In these cases, painful emotional experiences may remain disconnected from their source and meaning, and therefore persist active only within the subsymbolic system. In this way, the disconnection becomes recursive and potentially long-lasting. Consequently, within individuals’ interpersonal backgrounds, particularly among those with pronounced emotional and interpersonal inflexibility, specific and painful emotion schemas are formed in the individuals and are consistently activated at the subsymbolic level, which may remain disconnected from the symbolic levels. Disconnected emotion schemas are prototypical representations of self in relation to others, formed through repetition of interactions with painful affective states (Bucci 1997, 2021a) and include mainly subsymbolic elements (sensory, visceral, and motor). In this model, psychological suffering or psychopathology is seen as involving experiences of disconnection between the individual’s processing systems (disconnected emotion schemas subsymbolically recurrently reactivated) in interpersonal relationships with significant others leading to difficult and dysfunctional interactions that do not access symbolic levels.

Psychotherapy represents a special relationship with a human being – the therapist – who has achieved practical and theoretical competencies to interact on subsymbolic and symbolic levels with patients to reactivate their RP that could not fully develop in their previous history. The therapist’s action does not aim to change the patient or eliminate symptoms, but to ensure the conditions in which the RP of patient and therapist can be improved and enhanced, in order for the emotion schemas to become organized in a different, more integrated form. In this context, patients will find ways to live better with themselves and their worlds.

A good therapy session would entail the activation of the RP through phases of arousal, symbolizing, and reflecting/reorganizing. During the arousal phase, the patient grapples with an activation of sensory and bodily aspects of emotion schemas, struggling with the associated painful sentiments and thoughts. In the symbolizing phase, the patient begins articulating their feelings through imagery, words, and narratives, drawing from events, memories, dreams, or fantasies. Finally, with the support of therapeutic interventions, patients proceed to a phase of reflection and reorganization, broadening the interpretations of their experiences and fostering opportunities for novel connections and configurations in their emotion schemas (Bucci, 2021a; Bucci et al., 2016; Bucci & Maskit, 2007; Kingsley, 2010; Kris, 1956).

Bucci’s theoretical framework offers a lens through which therapeutic change can be understood as a dynamic process deeply intertwined with body, language, and social interaction. In therapy, patients are engaged in a multiformat and multifaceted exchange with the therapist, giving shapes, images, and words to their subjective experience. According to Bucci, psychotherapy is not only a verbal communication, but a complex intersubjective process through which patients, with their therapists, construct and reshape their understanding of themselves and their experiences. As patients verbalize their thoughts and feelings, they gain new insights, reframe their perspectives, and develop more adaptive ways of coping with daily life challenges. Moreover, within the therapeutic context, the therapist serves as a facilitator of this process, becoming an embodied partner in the relationship in which patients feel safe to explore their inner worlds. Through empathic listening, reflective questioning, and collaborative exploration, therapists help patients to elucidate and make sense of their experiences, fostering self-awareness, reorganizing the emotion schemas and creating new ones, and thus promoting personal growth. By attending to the ways in which patients construct meaning through dialogue and interpersonal exchange, therapists can facilitate profound shifts in understanding and behavior, ultimately promoting healing and change. Therapists themselves become elements in the new emotion schemas that are formed through the therapeutic relationship. This process enables patients to enhance their connections with their inner and interpersonal worlds, fostering deeper self-understanding and facilitating therapeutic progress. In this sense, the therapeutic relationship represents the main tool of the psychological intervention, but at the same time becomes the object of the intervention and can be understood as a means of first experiencing and then decoding the patient’s inner and relational experience.

Overall, MCT is grounded on a complexity-oriented epistemology. It is in fact an essentially psychological theory of human subjectivity understood as a complex process of connection with the world that generates a multiplicity of meanings that must be investigated systemically and from multiple levels and points of view. However, this investigation can by its nature only be done partially, to a limited degree. MCT is a theory of the human representational process rather than of behavior or the neurobiological functioning of the brain. The theory does not renounce seeking consistency with these two planes, but does not reduce the explanation of psychological phenomena to them. MCT values subjectivity as a tool of knowledge, proposes hypothetical and contextual assumptions rather than absolute and certain laws of function, and adopts complex and systemic explanatory models (see Table 1). Furthermore, it considers the dimensions and formats of the therapeutic relationship as the focus and goal of psychotherapy. Patient and therapist gradually become experts of these dimensions and in this way, they activate a process of change that is not predefined, but open to multiple outcomes and developments (see Table 2).

## Linguistic Measures of Referential Process and Complexity Epistemology

In recent decades, Bucci and colleagues have developed and validated some computerized linguistic measures of the RP functions (Bucci, 2021c; Mariani et al., 2013; Maskit, 2021; Negri et al., 2018; Zhou et al., 2021). These tools enable not only researchers, but also therapists, to observe, track, and quantify these functions, assessing in this way the dynamics of emotional elaboration occurring within the psychotherapeutic process.

The three main functions of the RP measured by these linguistic measures are the Arousal function (AF), the Symbolizing Function (SF), and the Reflecting/Reorganization Function (RRF).

When material outside or on the periphery of awareness is activated in the speaker, AF takes space (Bucci, 2021b, c; Tocatly, in this issue). The interpersonal encounter has the potential to activate the individual’s emotion schemas and this, to varying degrees, begins to fragmentarily connect emotion with images and words. This process may later find translation to a more consistent narrative.

SF is the speaker’s capacity to connect subsymbolic experiences to imagery and language and to communicate them effectively to the listener (Bucci, 1997, 2021b, Bucci, 2021c; Bucci et al., 2016). In other words, SF is the capacity of the speaker or writer to render their emotional, visceral, and relational experiences into words to engender related experiences in the listener or reader. Some characteristics of linguistic style reveal this capacity: the more the speech is concrete, specific, clear, and vivid, the more effectively words can convey the subsymbolic experience and perhaps activate corresponding experience in the listener (or reader).

RRF is the speaker’s capacity to see and find personal meanings and insights within their narrative, dream, or fantasy (Negri et al., 2018; Zhou et al., 2021). This does not refer to abstract or logical reasoning, but rather recognition of the subjective meaning of a memory in which the speaker is emotionally involved. There are some linguistic features revealing this reflective and reorganization function that can be measured.

Additional and complementary measures have been developed to investigate further aspects of RP, such as dictionaries of words related to disfluencies, affects, sensory and somatic sensations, and abstract reflection. The Discourse Attribute Analysis Program (DAAP) is the computer software (Maskit & Murphy, 2011) that applies these measures to any type of transcribed speech, producing quantitative indices and graphs that indicate the proportion in which those words are present in the text or the average weight they take on a specific construct.

Finally, there are some measures derived from the above that reveal the associations between the RP functions or some aspects of clinical exchange that are very important for understanding change processes. For example, one of these is the covariation between SF and RRF. If negative, this covariation indicates that the speaker alternates between vivid narration with high SF and reflection on it in which high RRF emerges. This negative covariation indicates the presence of a good elaborative process, associated with positive therapeutic outcomes (Bucci & Maskit, 2007; Mariani et al., 2013; Mariani & De Coro, 2013; Maskit, 2021).

Through these computerized linguistic measures, researchers and clinicians can measure and graphically monitor the progress of RP functions in a psychotherapy session or in an entire treatment; this allows detection of the presence or absence of the three phases that alternate, according to Bucci, in a good session: arousal, symbolization, and reflection/reorganization. The first phase is characterized by high AF and low SF, many disfluencies, and low RRF; in the symbolizing phase, SF is high while RRF and disfluencies are low; the reflecting/reorganizing phase shows low AF and SF, and high RRF (Bucci, 1997, 2021a, b, c; Maskit, 2021). This possibility of quantitatively and graphically representing the progress of the psychotherapeutic process through the application of the linguistic measures of RP have not only been widely used in psychological research contexts (Bucci, 1997a, 2021a, b, c; Bucci et al., 2016; Christian et al., 2021; Esposito et al., 2019; Gennaro et al., 2022; Mariani et al., 2013; Negri et al., 2024; Negri, Andreoli, Barazzetti, Negri et al., 2020a, b; Negri, Castiglioni, et al., 2022a, b; Negri & Christian, 2022; Negri, Christian, Mariani et al. 2019b; Negri & Ongis, 2021; Vegas et al., 2015) but have also served as a bridge between research and clinical practice worlds. In fact, they can add an external point of view on the psychotherapeutic process that complements the internal subjective ones of the therapist and patient’s accounts of the process itself. Linguistic measures can thus represent an additional tool, useful for clinical reasoning and/or supervision. For example, the trends in SF and RRF indices, and their covariation, as we have seen, can help the therapist to discern the possible beginnings of transformations that the therapeutic relationship is undergoing or to focus attention on some passages that may not have seemed significant at first glance. Moreover, discussion of cases in supervision and linguistic analysis of therapeutic interaction through linguistic measures are two moments that can effectively integrate, precisely because they provide information on the progress of the therapeutic process from different perspectives (Jaffe et al., 2024; Mariani & Negri, 2015). Other studies have used the linguistic measures in the context of psychotherapy – for example, analyzing the therapist’s notes (Bucci et al., 2012; Hoffman et al., 2013; Mariani & Negri, 2015; Negri, Andreoli, Mariani et al., 2020), and, in this way, giving the therapist’s subjective view of the psychotherapy process a meaningful and useful informant status.

These examples underscore that language measures can not only thoroughly investigate the therapeutic conversation by researchers, but also add a valuable external perspective for the participants involved in the psychotherapy process. Linguistic measures can serve as a crucial “third perspective” between patient and therapist, providing the therapist with an intuitive and beneficial routine resource in their clinical practice. When various points of view – such as those of the patient, therapist, linguistic measures, systematic observations from clinical and extra-clinical contexts – communicate and compare with each other in observing reality, we can more easily and reliably decide on the most plausible hypotheses about that reality, and in our case, about the therapeutic exchange. This multidimensional approach not only enriches the scientific understanding of the therapeutic process, but also enhances the accuracy and depth of clinical insights. Therefore, the use of linguistic measures of the RP can be understood not as an objective measurement tool, but as a rudder to navigate within human relationships, integrating different points of view, levels of observation, and data from different contexts and meaning systems.

In sum, we can view the linguistic measures of the RP as providing a means of observation based on a complexity epistemology and bridging the research and clinical intervention perspectives (See Table 3). In fact, they can be used as flexible, reflexive, and intersubjective practices within the ecological context of the psychotherapy to grasp a plausible and situated knowledge; they are aimed at investigating the interaction of the multiple variables, including the researchers’ influences, valuing the uniqueness and subjective points of view, broadening the observation field to systemic explanations; and finally, they can add a third external source of information to those represented by the patient’s and therapist’s internal points of view. The application of linguistic measures of the RP to the psychotherapy session or treatment, in fact, makes it possible to indirectly measure how even the judges involved in the construction of the linguistic measures – which evaluated many sessions relating to the main functions of RP – might perceive and evaluate the discourse under examination. In this sense, the linguistic measures of RP adds to and dialogues with the views of researchers, patients, and therapists (Jaffe et al., 2024). This process represents a unique methodological triangulation of viewpoints on the same emotional communication processes which allows to identify, through the comparison of the various sources, the most plausible and intersubjectively shared hypotheses and, thus, to have a more realistic and complex view of the psychotherapeutic process.

## Conclusion

Adopting a complexity epistemology is crucial when considering our object of inquiry as researchers and practitioners of psychology: the human subjectivity. Complexity epistemology emphasizes the importance of multiple levels and points of observation in determining which hypotheses are most plausible for explaining reality. It therefore recognizes the specificity of its object of study, which takes shape and can only be investigated through intersubjective encounters, adapting its methods, theories, and practices to make them consistent with these specificities.

MCT is consistent with this epistemology by viewing the mind not as a fixed set of internal states, but as an ongoing relational process between the subject and the world. Incorporating linguistic measures into a complexity epistemology provides a solid foundation for comprehending the intricate and ever-changing dynamics of human mentation. This perspective highlights the relational, embodied, and multifaceted dimensions of the therapeutic exchange, enriching our capacity to understand the subtle processes inherent in psychological growth and adjustment. Therefore, utilizing the linguistic measures of RP allows researchers and clinicians to delve deeper into the mechanisms driving therapeutic change, facilitating the development of interventions that are more impactful and tailored to individual needs.

The concept of the RP, central to MCT, is consistent with the notion that the human mind incorporates continuous and multiple processes. RA and RRF measure how effectively experiences are articulated and understood within the therapeutic dialogue. If therapeutic exchange aims to activate and reorganize emotion schemas, linguistic measures capture these shifts, providing insights on how patients process and integrate their symbolic and subsymbolic levels of experiences. Thus, the therapeutic process is seen as a flexible and open system.

Using linguistic measures of RP in psychotherapy and research can be a practice based on a complexity epistemology in that it allows the tracking of the dynamic interplay among different levels of processing (subsymbolic, non-verbal symbolic, verbal symbolic) and among different points of view (patients, therapists, linguistic computerized analysis). The measures also emphasize the flexibility, responsiveness, and reflectiveness of the human systems under investigations. Furthermore, linguistic measures are grounded in complexity epistemology by investigating communication and meaning-making processes, rather than focusing solely on behaviors. They provide a distinct perspective on the reality of therapeutic exchanges. By comparing data from various points of observation, we can develop more plausible hypotheses about therapeutic exchanges. All viewpoints contribute slightly different information, and none has the absolute truth or correct view of reality. However, when these viewpoints communicate and complement each other in observing reality, it increases our reliability in choosing the most plausible hypotheses regarding human complexity.

## Footnotes

1 The Age of Enlightenment was a cultural movement that occurred in Europe in the 17th and 18th centuries. The term referred to the “enlightenments” of reason and science as tools to combat the ignorance and superstition of previous centuries. Key features of this movement were unconditional faith in the potential of human reason and faith in nature. In particular, faith in rationality led to the application in the field of knowledge of the experimental method, with the conviction that this method could identify general laws and arrive at the reconstruction of a new era of happiness and well-being.
